# Laboratory Evaluation of the DPP Syphilis Screen & Confirm Assay

**DOI:** 10.1128/spectrum.02642-21

**Published:** 2022-05-31

**Authors:** Silver K. Vargas, Jazmin Qquellon, Francesca Vasquez, Kelika A. Konda, Gino Calvo, Michael Reyes-Diaz, Carlos Caceres, Jeffrey D. Klausner

**Affiliations:** a Center for Interdisciplinary Studies in Sexuality, AIDS, and Society, Universidad Peruana Cayetano Heredia, Lima, Peru; b Division of Infectious Diseases, David Geffen School of Medicine, University of California, Los Angeles, Los Angeles, California, USA; c Department of Population and Public Health Sciences, Keck School of Medicine, University of Southern California, Los Angeles, California, USA; University of Cincinnati

**Keywords:** dual testing, point of care, immunoserology, syphilis

## Abstract

Because syphilis is a public health concern, new strategies and tools for detecting active syphilis cases should be evaluated for future implementation. We assessed the laboratory performance of the DPP Syphilis Screen & Confirm rapid immunodiagnostic test (Chembio Diagnostics, Medford, NY, USA), using visual reading and the manufacturer’s electronic test microreader, for detection of treponemal and nontreponemal antibodies in 383 fully characterized stored serum specimens. We used the Treponema pallidum particle agglutination (TPPA) test and rapid plasma reagin (RPR) test as reference tests for the DPP Syphilis Screen & Confirm assay treponemal and nontreponemal components, respectively. The sensitivity values for treponemal antibody detection by electronic reader and visual interpretation were 83.2% and 85.9%, respectively, with 100% specificity. For nontreponemal antibody detection, the sensitivity values were 65.7% and 69.0% and the specificity values were 88.7% and 89.4% for electronic reader and visual interpretation, respectively. There was excellent correlation between visual interpretation and the microreader for either component (kappa coefficient, 0.953). When restricting the analysis to RPR titers of ≥1:8, the sensitivity was 96.9% for either reading method; numerical microreader values showed good correlation with RPR titers (Spearman rho of 0.77). The DPP Syphilis Screen & Confirm assay showed good performance, compared to reference syphilis tests, using serum. Field evaluation studies should be done to validate its use for detection of active cases and for monitoring of treated syphilis patients.

**IMPORTANCE** Syphilis remains a public health problem; therefore, health systems must incorporate screening tools that allow a rapid and accurate diagnosis to provide adequate treatment. The DPP Syphilis Screen & Confirm Assay simultaneously detects treponemal and nontreponemal antibodies, emerging as an alternative for identifying cases in situations in which there is no infrastructure to perform conventional syphilis testing, but it is necessary to generate evidence regarding the performance of this technology in various scenarios. We found that the test performs well, compared to TPPA and RPR tests, using stored samples from participants at high risk of acquiring syphilis. Additionally, when the Chembio microreader was incorporated, similar results are obtained by the device, compared to those reported by trained laboratory professionals, and correlated with the semiquantitative results of the RPR test. We think that the use of the DPP Syphilis Screen & Confirm Assay with the microreader might help in detecting active syphilis cases and perhaps in monitoring treatment responses in the field.

## INTRODUCTION

Syphilis, which is caused by the spirochete Treponema pallidum subsp*. pallidum*, remains a public health concern. At least 40% of the countries reporting to the Global AIDS Monitoring system estimated that more than 5% of men who have sex with men (MSM) were infected by syphilis in 2019, with the region of the Americas having the highest median prevalence (12.4%) (1). In Peru, the prevalence of syphilis in high-risk MSM was estimated at up to 20.0% (2, 3), while the prevalence in transgender women was 22.9% (4).

Laboratory diagnosis of syphilis is mainly through serological assays that detect nontreponemal and treponemal antibodies. Both types of tests are needed to identify active syphilis cases and are performed as consecutive steps or in testing algorithms (5). For years, syphilis screening was done using a nontreponemal antibody test like the rapid plasma reagin (RPR) test or the Venereal Disease Research Laboratory (VDRL) test, and Treponema pallidum particle agglutination (TPPA) assay or fluorescent treponemal antibody absorption (FTA-ABS) tests were used as confirmatory tests; this was called the traditional algorithm. However, an alternative algorithm uses a treponemal antibody test, like an enzyme-linked immunoassay (ELISA) or chemiluminescence immunoassay (CLIA), for initial screening, followed by a nontreponemal test for the reactive specimens only, and may include a second treponemal antibody assay if the nontreponemal test results are negative, to exclude false-positive treponemal antibody results (6).

Irrespective of the algorithm used, nontreponemal antibody tests like the RPR and VDRL assays are used as semiquantitative tests (antibody titration) for serological follow-up after completing treatment; evidence of a 4-fold decrease in titer is interpreted as successful treatment (7). When the nontreponemal antibody titer does not decrease 4-fold after 12 months of treatment, individuals might be considered a treatment failure or serofast (7, 8). The prevalence of serological nonresponders was estimated as 20.5% at 6 months after receiving treatment and decreased to 11.2% at ≥12 months, for all stages of syphilis (8). Sexually transmitted disease treatment guidelines recommend posttreatment follow-up evaluation, although it is often difficult because people do not return in a timely manner to evaluate whether the treatment received was effective (9). The major constraints of both algorithms had to do with the need for trained personnel for performing the tests and the need for equipment for both treponemal and nontreponemal antibody assays, which restricts dissemination and implementation of conventional syphilis screening to laboratories with at least basic infrastructure (6). To overcome these limitations, a great number of syphilis point-of-care (POC) tests have been developed (10) and evaluated in laboratories (11, 12) and clinical sites (13), showing good performance in comparison with conventional laboratory-based assays. While those POC tests improve health access for target populations (14, 15), most of them are designed for detection of treponemal antibodies only, which makes the identification of active cases difficult, given that treponemal antibodies persist after successful treatment. For that reason, there is a persistent need for other assays that detect nontreponemal antibodies. Additionally, there are no easy-to-use rapid POC tests for detection of changes in serological titers to monitor treatment success. Therefore, there is a substantial need for assays that can simultaneously detect the presence of treponemal and nontreponemal antibodies, such as the DPP Syphilis Screen & Confirm assay (16).

In this study, we evaluated the performance of the DPP Syphilis Screen & Confirm assay for the simultaneous detection of treponemal and nontreponemal antibodies, using deidentified stored serum specimens collected from people at high risk for syphilis.

## RESULTS

In total, 383 sera were tested, of which 79 were TPPA negative and RPR nonreactive, 62 were TPPA positive and RPR nonreactive, and 242 were TPPA positive and RPR reactive, with RPR titers from 1:1 to 1:256. Clinical information on syphilis status was as follows: 95 samples (24.8%) were from participants without syphilis, 263 samples (68.7%) were from individuals with treated syphilis, and 25 samples were from patients with active syphilis (6.5%). Also, 204 (53.3%) of the 383 samples were from individuals with HIV-1 infection. Due to the sample storage, we repeated the RPR test on 20 randomly selected samples; 18 showed the same RPR result as the initial finding, while 2 samples had a 1 dilution decrease.

For the treponemal antibody component, the sensitivity and specificity were 85.9% (95% confidence interval [CI], 81.4% to 89.6%) and 100.0% (95% CI, 95.4% to 100.0%), respectively, for visual reading (Table 1). When using the Chembio microreader qualitative results (cutoff value of 9.0), the sensitivity and specificity were 83.2% (95% CI, 78.5% to 87.2%) and 100.0% (95% CI, 95.4% to 100.0%), respectively.

**TABLE 1:** Performance of DPP Syphilis Screen & Confirm with visual operator inspection and microreader analysis (cutoff of 9.0) against reference comparator tests

Assay and detection method^*a*^	No. of samples with:		Agreement (95% CI) (%)	Sensitivity (95% CI) (%)	Specificity (95% CI) (%)	Kappa coefficient (95% CI)
	True-positive results	True-negative results				
Chembio treponemal component	*n* = 304	*n* = 79				
Operator reading	261	79	88.7 (85.6–91.9)	85.9 (81.4–89.6)	100.0 (95.4–100.0)	0.71 (0.63–0.79)
Microreader	253	79	86.6 (83.2–90.1)	83.2 (78.5–87.2)	100.0 (95.4–100.0)	0.67 (0.59–0.75)
Chembio nontreponemal component	*n* = 242	*n* = 141				
Operator reading	167	125	76.2 (71.9–80.5)	69.0 (62.8–74.8)	88.7 (82.2–93.4)	0.53 (0.44–0.61)
Microreader	159	126	74.4 (70.0–78.8)	65.7 (59.4–71.7)	89.4 (83.1–93.9)	0.50 (0.41–0.58)

a Reference tests were the TPPA test for treponemal antibodies and the RPR test for nontreponemal antibodies.

For the nontreponemal antibody component, the sensitivity and specificity for visual reading were 69.0% (95% CI, 62.8% to 74.8%) and 88.7% (95% CI, 82.2% to 93.45%), respectively; with the microreader (cutoff value of 9.0), the sensitivity and specificity were 65.7% (95% CI, 59.4% to 71.7%) and 89.4% (95% CI, 83.1% to 93.9%), respectively. The kappa coefficient for visual reading versus microreader qualitative results for the treponemal antibody component was 0.95 (95% CI, 0.92 to 0.99) and that for the nontreponemal antibody component was 0.95 (95% CI, 0.92 to 0.98) (data not shown).

There was no statistical difference in the detection of treponemal antibodies according to HIV infection status for both operator readings nor microreader results (*P* values of > 0.1) (Table 2).

**TABLE 2:** Performance of DPP Syphilis Screen & Confirm with visual operator inspection and microreader analysis (cutoff of 9.0) according to HIV infection status

Assay and detection method	No. of samples with:		Agreement (95% CI) (%)	Sensitivity (95% CI) (%)	Specificity (95% CI) (%)	Kappa coefficient (95% CI)
	True-positive results	True-negative results				
HIV-1 positive^*a*^						
Chembio treponemal component^*b*^	*n* = 165	*n* = 39				
Operator reading	138	39	86.7 (82.0–91.4)	83.6 (77.1–88.9)	100.0 (91.0–100.0)	0.66 (0.55–0.77)
Microreader	134	39	84.8 (79.8–89.7)	81.2 (74.4–86.9)	100.0 (91.9–100.0)	0.62 (0.51–0.73)
Chembio nontreponemal component^*b*^	*n* = 132	*n* = 72				
Operator reading	100	63	79.9 (74.3–85.4)	75.8 (67.5–82.8)	87.5 (77.6–94.1)	0.58 (0.47–0.69)
Microreader	96	63	77.9 (72.2–83.6)	72.7 (64.3–80.1)	87.5 (77.6–94.1)	0.55 (0.44–0.66)
HIV-1 negative						
Chembio treponemal component^*b*^	*n* = 139	*n* = 40				
Operator reading	123	40	91.0 (86.8–95.2)	88.5 (82.0–93.3)	100.0 (91.2–100.0)	0.77 (0.67–0.87)
Microreader	119	40	88.8 (84.1–93.4)	85.6 (78.7–91.0)	100.0 (91.2–100.0)	0.72 (0.61–0.83)
Chembio nontreponemal component^*b*^	*n* = 110	*n* = 69				
Operator reading	67	62	72.0 (65.4–78.7)	60.9 (51.1–70.1)	89.9 (80.2–95.8)	0.46 (0.34–0.58)
Microreader	63	63	70.3 (63.6–77.1)	57.3 (47.5–66.7)	91.3 (82.0–96.7)	0.43 (0.32–0.55)

a The HIV-1 confirmatory test was Western blotting.

b Reference tests were the TPPA test for treponemal antibodies and the RPR test for nontreponemal antibodies.

**FIG 1:**
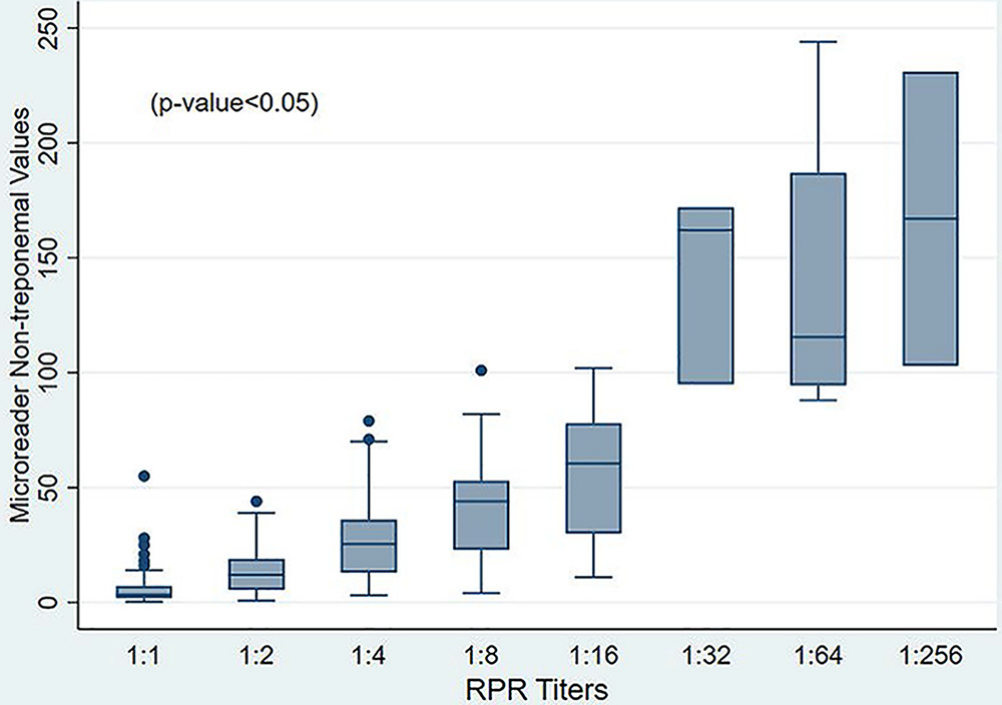
Distribution of microreader values of nontreponemal component according to RPR titer.

Nontreponemal microreader median values by RPR titer are shown in Table 3. Median microreader values for RPR-negative and RPR 1:1 samples were 2.8 (interquartile range [IQR], 1.8 to 4.2) and 3.4 (IQR, 1.9 to 7.2), respectively, below the default cutoff value of 9.0; samples with a 1:8 RPR titer had a median microreader value of 44 (IQR, 23 to 53). When analyzing sensitivity of nontreponemal component by RPR titer, we found a sensitivity of 58.5% (95% CI, 50.9 to 65.9%) and 54.0% (95% CI, 46.3 to 61.5%) for RPR titers up to 1:4 with visual reading and microreader qualitative results, respectively. A sensitivity of 96.9% (89.5 to 99.6%) was found for RPR titers of 1:8 or higher with both reading methods (Table 4). Additionally, we found a good correlation (Spearman's rho of 0.77) between the Chembio microreader numerical values and RPR titers. Finally, the nontreponemal microreader median values were different when analyzed by RPR titer (*P* < 0.05) (Fig. 1).

**TABLE 3:** Distribution of microreader nontreponemal values by RPR titer

RPR titer	No. of samples	Microreader nontreponemal value(s)		
		Mean	Median	IQR
Negative	141	4.4	2.8	1.8–4.2
1:1	71	6.3	3.4	1.9–7.2
1:2	59	13.5	12	5.5–19
1:4	46	27.2	25.5	13–36
1:8	37	43.0	44	23–53
1:16	20	56.1	60.5	30–78
1:32	3	143	162	95–172
1:64	4	140	115.5	94.5–187
1:256	2	167	167	103–231
Total	383	18.7	6.1	2.5–23

**TABLE 4:** Sensitivity of DPP Syphilis Screen & Confirm nontreponemal antibody component with visual operator inspection and microreader analysis (cutoff of 9.0)

RPR titer	Sensitivity (95% CI)	
	Operator result	Microreader result
Categorized RPR titers		
≤1:4	58.5 (50.8–65.8)	53.9 (46.3–61.5)
≥1:8	96.9 (89.4–99.6)	96.9 (89.4–99.6)
Detailed RPR titers		
1:1	26.7 (16.9–38.5)	19.7 (11.2–30.8)
1:2	71.1 (57.9–82.2)	66.1 (52.6–77.9)
1:4	91.3 (79.2–97.5)	91.3 (79.2–97.5)
1:8	94.5 (81.8–99.3)	94.5 (81.8–99.3)
1:16	100.0 (83.1–100.0)	100.0 (83.1–100.0)
1:32	100.0 (29.2–100.0)	100.0 (29.2–100.0)
1:64	100.0 (39.7–100.0)	100.0 (39.7–100.0)
1:256	100.0 (15.8–100.0)	100.0 (15.8–100.0)

## DISCUSSION

Because serological diagnosis of syphilis requires detection of treponemal and nontreponemal antibodies, there is a need for screening platforms that perform both evaluations faster, simultaneously, and easily. The DPP Syphilis Screen & Confirm assay showed good performance using stored serum samples. Additionally, the Chembio electronic microreader performed similarly to visual operator readings.

The treponemal antibody component showed better performance results than those reported by Zorzi et al. (17). In that study, the investigators used several biological samples and obtained sensitivity values between 57% and 64% for treponemal antibodies in serum. On the other hand, our results show a performance for the treponemal antibody component closer to that found by Causer et al. (18), who reported a sensitivity of 89%, but lower than values reported by other authors (12, 19, 20).

The nontreponemal component showed lower performance for qualitative RPR results as the reference comparator. When stratified by RPR titer, we found that the sensitivity of the DPP Syphilis Screen & Confirm assay nontreponemal component performed very well on sera with RPR titers of ≥1:8. Other studies reported similar findings when assessing samples with high RPR titers (18, 20, 21). Castro et al. reported that the majority of false-negative samples with the DPP Syphilis Screen & Confirm assay had RPR titers up to 1:2, but the sensitivity improved when those were excluded (16). Researchers also found that performance of the DPP Syphilis Screen & Confirm assay nontreponemal antibody component was related to syphilis stage (18, 22). Those findings may suggest that the DPP Syphilis Screen & Confirm assay could identify the majority of syphilis cases, given that most syphilis cases are detected with high RPR titers, suggesting active or untreated syphilis, whereas a low RPR titer may be related to previously treated infection, due to nontreponemal antibodies that decline after treatment (23). However, some cases of very early disease and late latent cases could be missed as individuals in these stages of syphilis infection may present with lower nontreponemal antibody titers. These considerations should be taken into account when implementing syphilis screening in programs that require frequent syphilis testing, like those for preexposure prophylaxis for HIV infection, where users are monitored quarterly and inaccurate results at lower RPR titers may result in missed new cases.

The Chembio electronic microreader reported qualitative results (cutoff of 9.0) equivalent to operator results; furthermore, microreader device numerical results showed good correlation with RPR titers. Good microreader performance was reported for this syphilis assay (20) and other dual tests (24). The electronic reader demonstrated performance equivalent to that of laboratory-trained staff members, and this could mean that the Chembio microreader might be used for active syphilis case detection and treatment monitoring when a conventional RPR test is unavailable or DPP Syphilis Screen & Confirm assay testing is performed by nonlaboratory health professionals. In the study of Langendorf et al. (25), the DPP Syphilis Screen & Confirm assay results were read visually by clinical staff members and laboratory professionals at a clinic in Burkina Faso. However, almost one-third of nontreponemal DPP Syphilis Screen & Confirm assay line results reported by the site laboratory professionals were missed by clinicians, which may be related to unfamiliarity with the assay or lack of training something that could be overcome if the microreader were used.

We acknowledge the following limitations in this study. We assessed the DPP Syphilis Screen & Confirm assay using stored sera. While most samples tested were directly stored at −80°C and were not exposed to frost-defrost cycles, long-term storage might have affected the titer of nontreponemal antibodies, mainly in low-titer RPR samples. In this context, we performed RPR tests for a random set of samples before DPP Syphilis Screen & Confirm assay evaluation, and titer results remained the same or varied only by 1 dilution, compared to the original results, making the samples suitable for this laboratory analysis. We included samples from people at high risk of syphilis, and we did not consider populations with low syphilis risk, such us pregnant women or the general population; therefore, we cannot ascertain the performance of the DPP Syphilis Screen & Confirm assay test among these populations. Additionally, highly trained staff members performed reference comparator tests and DPP Syphilis Screen & Confirm assay test procedures and thus the high level of concordance between operator and microreader results may be an overestimate; however, the microreader could perform better in a field trial with less-well-trained staff members. Finally, our results for the performance of the DPP Syphilis Screen & Confirm assay were somewhat lower than previously reported by other investigators and might be influenced by the study population or the manufacturing lot included in our analysis (20). Despite these limitations, the Chembio DPP Syphilis Screen & Confirm assay used with the microreader complies with REASSURED criteria for POC syphilis tests, which establish a sensitivity of at least 75% and a specificity of 92% for treponemal antibody detection; however, there are no criteria for nontreponemal POC tests (26).

Given the evidence of proven performance of POC syphilis tests (13, 23), such tests should be considered in the clinical algorithms for the diagnosis and treatment of syphilis in situations in which a single testing visit is preferable. Same-day testing and results might also help to reduce mistreatment and/or overtreatment caused by syndromic management (10). Furthermore, it would be a great tool for providing care to hard-to reach populations (27, 28). For the successful inclusion of new technologies (29), the performance characteristics of each assay and the capabilities of each health system must be taken into account so that the maximum benefit can be obtained with implementation (30, 31).

Finally, based on our results, we hypothesize that the DPP Syphilis Screen & Confirm assay could be used in the following algorithm: patients with a negative DPP Syphilis Screen & Confirm assay test result but high suspicion or risk for syphilis must be retested after 1 week, expecting a titer increase. In addition, in high-consequence populations such as pregnant women, repeated testing throughout the pregnancy should be recommended. However, there should be more studies evaluating the performance of the DPP Syphilis Screen & Confirm assay-microreader system in the laboratory with prospective samples, followed by studies focusing on the implementation of the DPP Syphilis Screen & Confirm assay along with the microreader to validate its utility in clinical practice in scenarios in which POC testing may have a great impact on syphilis management.

## MATERIALS AND METHODS

### Specimen collection.

Blood samples were collected between 2013 and 2016 from a cohort of MSM and transgender women attending sexually transmitted infection clinics in Lima, Peru (32). After collection of blood samples, the clinic laboratory staff members separated serum by centrifugation into several aliquots of 1 mL each for protocol procedures and long-term storage. Syphilis screening was performed for all samples using a treponemal antibody rapid test (Determine Syphilis; Alere, Israel) at clinic sites. The nontreponemal Macro-Vue RPR test (Becton, Dickinson, NJ) was also performed on all samples. Serum aliquots were stored at −20°C until adequate transportation to the Laboratory of Sexual Health at University Peruana Cayetano Heredia. Confirmatory syphilis testing was performed using the Serodia TPPA assay (Fujirebio Diagnostics Inc., Sweden) to fully characterize syphilis status for all samples collected, irrespective of their previous RPR or rapid test result. Additionally, HIV infection status was defined by Western blotting (New LAV blot 1; Bio-Rad, France). Finally, serum samples were stored at −80°C for future research. For this evaluation, we repeated RPR testing for 20 samples in March 2021 to evaluate the effect of storage on serum antibody levels.

### DPP Syphilis Screen & Confirm assay.

The DPP Syphilis Screen & Confirm assay was performed at the Laboratory of Sexual Health, Universidad Peruana Cayetano Heredia (Lima, Peru), using the stored serum samples following the manufacturer’s instructions. First, 5 μL of serum was added to the sample well of the test device; then, 2 drops of kit running buffer were added to the sample well. After 5 min, 2 drops of the kit running buffer were added to well 2. After 15 min, one trained staff member read the tests results visually and recorded them. Next, we used the Chembio microreader device (catalog number 70-1000-1), which scans the DPP test cartridge and displays a numerical value based on test line color intensity, to obtain the quantitative values for the treponemal and nontreponemal test lines and result interpretation. Instructions for using the microreader are the following. First, place the reader in its holder, and press the button to turn the device on. Then, press the button again and a radiofrequency identification (RFID) display appears, which means we need to place the RFID card that contains information on the assay on top of the reader. After removing the RFID card, the word TEST appears; next, we need to press the button again so that the reader displays the word RUN, and finally we scan the DPP cartridge and finally the microreader displays a numerical value and interpretation for each test line. For qualitative results, default cutoff values of the microreader device were as follows: nonreactive, <9.0; reactive, ≥9.0.

### Statistical analysis.

For our analysis, we evaluated the performance of the DPP Syphilis Screen & Confirm assay for detection of treponemal and nontreponemal antibodies by visual reading and with the microreader cutoff results, using the TPPA test as reference for the treponemal antibody component and the RPR assay for the nontreponemal antibody component. We estimated the sensitivity and specificity for each assay component. We used the exact binomial method to determine 95% CIs and estimated medians and 25th and 75th percentiles of microreader numerical values by RPR titer for the nontreponemal component. We also calculated concordance between the DPP Syphilis Screen & Confirm assay and the respective reference tests and concordance between visual results and the microreader qualitative results (cutoff of 9.0) using Cohen’s kappa statistic. In addition, we assessed the performance stratified by HIV infection status.

We also assessed the performance of the DPP Syphilis Screen & Confirm assay for detecting nontreponemal antibodies by RPR titer using sensitivity results from visual reading and microreader evaluation by cutoff. We then used the Spearman correlation coefficient to evaluate the correlation between the Chembio electronic microreader numeric nontreponemal component results and the RPR titer. We finally used the Kruskal-Wallis test to evaluate the distribution of nontreponemal component microreader values by RPR titer. All analyses were conducted using Stata v.16 (College Station, TX, USA).
